# Differential Effects of Intrauterine Balloon Tamponade Indications on Postpartum Hemorrhage Outcomes

**DOI:** 10.3389/fmed.2022.851174

**Published:** 2022-04-01

**Authors:** Chuangchuang Xu, Yiyao Chen, Lin Wen, Xiaolei Chi, Xinliang Chen

**Affiliations:** ^1^Department of Obstetrics and Gynecology, International Peace Maternity and Child Health Hospital, School of Medicine, Shanghai Jiao Tong University, Shanghai, China; ^2^Department of Pelvic Floor Clinic Center, International Peace Maternity and Child Health Hospital, School of Medicine, Shanghai Jiao Tong University, Shanghai, China; ^3^Shanghai Key Laboratory of Embryo Original Disease, Shanghai Jiao Tong University, Shanghai, China

**Keywords:** postpartum hemorrhage, placental site bleeding, uterine atony, intrauterine balloon tamponade, blood transfusion, estimated blood loss, uterine artery embolization, hysterectomy

## Abstract

**Objective:**

To determine whether the indication of intrauterine balloon tamponade (IUBT) was associated with postpartum hemorrhage (PPH) outcomes.

**Methods:**

Patients with PPH who underwent IUBT between January 2013 and November 2021 were included in a cohort study. PPH outcomes in patients who had IUBT for indications of uterine atony were compared to those who had IUBT for indications of placental site bleeding. PPH outcomes included uterine artery embolization (UAE) or hysterectomy after IUBT, estimated blood loss (EBL) after balloon placement, and blood transfusion. Statistical analysis was performed using multivariate logistic regression.

**Results:**

IUBT was performed on 603 cases, with 121 (20.1%) undergoing it for placental site bleeding and 482 (79.9%) for uterine atony. In general, IUBT was a reliable treatment for PPH, but the specific efficacy varied depending on the indication for placement. After controlling for confounding variables, compared to the indication of uterine atony, the indication of placental site bleeding increased the risk of transfusion of ≥4 units of PRBCs (aOR 2.47, 95%CI 1.32–3.98), EBL ≥ 300 ml after IUBT (aOR 3.78, 95%CI 2.22–5.33), and UAE or hysterectomy (aOR 2.52, 95%CI 1.20–6.01), respectively. Other factors associated with adverse PPH outcomes were lower antenatal hemoglobin, higher IUBT volume, longer duration of IUBT and larger shock index values.

**Conclusions:**

IUBT was less effective in treating PPH patients with indications of placental site bleeding than with indications of uterine atony. Follow-up monitoring of PPH patients with placental site bleeding should be intensified.

## Introduction

Postpartum hemorrhage (PPH) remains the primary source of maternal morbidity and mortality worldwide, accounting for nearly a third of all maternal deaths (1, 2). The number of elderly pregnant women and parous women in China has increased dramatically since the implementation of the two-child policy in 2005 (3). Accordingly, the proportion of patients with PPH has also increased, which poses a great challenge for postpartum management (4).

PPH requires multistep treatment. Invasive second-line management, such as intrauterine suture, uterine artery embolization (UAE), and uterine vessel ligation, may be attempted after a first-line treatment, such as uterotonics, has failed (5, 6). If none of these options is viable, a hysterectomy can be performed as a life-saving procedure (7). However, the adverse effects on fertility associated with invasive procedures and hysterectomy prevent their use from being the preferred method.

Intrauterine balloon tamponade (IUBT) was first used to treat PPH by Bakri et al. (8) in 1992, and numerous studies have confirmed the efficacy of IUBT in treating PPH while avoiding the problem of reduced fertility due to invasive procedures (9–11). Despite its widespread use today, IUBT is still largely empirical and devoid of standardization. Specifically, to the best of the authors' knowledge, the relationship between IUBT placement indications and PPH–related outcomes remains unidentified.

Thus, we examined up to 9 years of total cases at the institution to investigate the effect of different IUBT indications on PPH outcomes as well as other factors associated with PPH outcomes. We hypothesized that women with IUBT indications of placental site bleeding would have worse PPH outcomes than women with IUBT indications of uterine atony.

## Materials and Methods

### Study Design and Patients

This is a cohort study conducted at the International Peace Maternal and Child Health Hospital (IPMCH), affiliated with Shanghai Jiao Tong University. The clinical data of all women who had IUBT for severe PPH at the institution between January 2013 and November 2021 were analyzed retrospectively. According to the International Federation of Gynecology and Obstetrics (FIGO) guidelines, a total estimated blood loss (EBL) ≥ 1,000 ml was defined as severe PPH, with specific values recorded in the critical care record form of the hospital electronic medical record. IUBT is the main option for the rescue of refractory postpartum hemorrhage (ineffectiveness of combined uterine massage with uterotonics) in the study institution. Intrauterine suture or B-Lynch suture may have been performed to stop bleeding prior to IUBT for cesarean delivery. The indications for IUBT are determined by the obstetrician and include uterine atony and placental site bleeding. If the obstetrician documented the indication as uterine atony or if the medical record described the cause of bleeding as softness of the lower uterine segment or softness of the uterine global, the main indication was defined as uterine atony. If the documented indication was placental site bleeding, or if the main description in the medical record was about placental abnormalities (placental invasion, or placenta residue), or if the bleeding point was clearly indicated to be located in the placental bed, the main indication was defined as placental site bleeding.

We created a subset cohort of eligible women who performed IUBT based on the following exclusion criteria: underwent IUBT due to placental site bleeding combined with uterine atony, IUBT was inserted when PPH < 1,000 ml, and performed prophylactic IUBT due to high-risk delivery factors (e.g., central placenta previa, coagulation disorders, uterine malformation, etc.).

### IUBT Placement and Removal

The IUBT device used in the institution was the Bakri balloon (Bakri Medical, Bloomington, USA). According to the cervical opening, the IUBT balloon was inserted through the cesarean incision or vagina. When the IUBT balloon was inserted through the cesarean incision, a volume of saline was first flushed in based on the operator's estimated size of the uterine cavity to prevent dislodgement during the operation. After suturing the incision, saline was flushed again in under ultrasound guidance until the blood flow through the device catheter was reduced significantly. When the IUBT balloon was inserted through the vagina, a one-time flush of saline is applied to the balloon if the ultrasound observes that the balloon is in the correct position. After the operation, a large piece of gauze approximately 10 cm wide was filled into the vagina to prevent the balloon from falling out of its normal position. The time of IUBT placement and the total volume of saline flushed into the balloon are recorded. After IUBT, the patients were monitored in the monitoring ward for a period of time, which included blood pressure, heart rate, and other vital signs, as well as assessment of ongoing blood loss. The duration of IUBT was decided by the physician based on the patient's condition and EBL and usually takes no more than 24 h. Prior to the IUBT balloon removal, 20 U of oxytocin was administered intravenously. During the procedure, 50 mL of saline was withdrawn from the IUBT balloon every 15 min with assisted uterine massage, and the balloon and vaginal gauze were withdrawn if there was no active bleeding.

### Data Collection

All study data was obtained from electronic cases. Demographic and obstetric data included maternal age at delivery (years), pre-pregnancy BMI (kg/m^2^), nulliparous (yes, no), multiple gestation (yes, no), premature birth (yes, no), induction of labor (yes, no), history of PPH (yes, no), antenatal hemoglobin (g/dl), delivery mode (cesarean, vaginal). IUBT-related data included: surgical hemostasis before IUBT (none, intrauterine suture, B-Lynch suture), uterotonics before IUBT (0 or 1, ≥2), time from birth to IUBT (min), IUBT volume (<250, 250–349, ≥350 ml), duration of IUBT (hours), postpartum shock index (SI; calculated as the ratio of heart rate to systolic blood pressure) (<1, 1–1.4, ≥1.5).

### Outcomes

PPH outcomes included UAE or hysterectomy, EBL ≥ 300 ml after IUBT, and transfusion of ≥4 units of packed red blood cells (PRBCs). Intrauterine suture and B-Lynch suture were not included, as these measures may have been used before IUBT. UAE implemented prior to IUBT is not counted. The output from the IUBT device itself, the weight of disposable pads and drapes, and the number of blood-soaked sponges were all used to calculate EBL after IUBT. All caregivers of PPH patients have been trained in standardized bleeding estimation.

### Statistical Methods

Distributions of maternal demographic, obstetric, and IUBT-related data were shown as numbers (percentages) or mean ± SD or median and interquartile range. The chi-square test was used to compare categorical variables and the independent sample *t*-tests or Mann-Whitney *U*-tests were used to compare continuous variables. Crude ratios (OR) and 95% confidence intervals were calculated for all factors studied in the analysis. Univariate and multivariate logistic regression analyses were performed to identify independent prognostic factors for each PPH outcome. We examined potential confounders for multivariate modes based on a literature review and included factors from univariate analysis with *p-*values < 0.20. The following variables were included in the multivariate logistic regression analysis: maternal age, multiple gestation, antenatal hemoglobin, delivery mode, time from birth to IUBT, IUBT volume, duration of IUBT, postpartum SI.

All data was processed using SPSS 25.0 (Windows, IBM, Armonk, NY, USA). Statistical significance was defined as *p-*values < 0.05.

### Ethics

The protocol was approved by the IPMCH Ethics Committee and the requirement for informed consent was waived (GKLW 2021–55). Since our study is a retrospective cohort study, clinical trial registration is not required. All procedures followed were carried out in accordance with the Helsinki Declaration's ethical standards.

## Results

**Figure 1:**
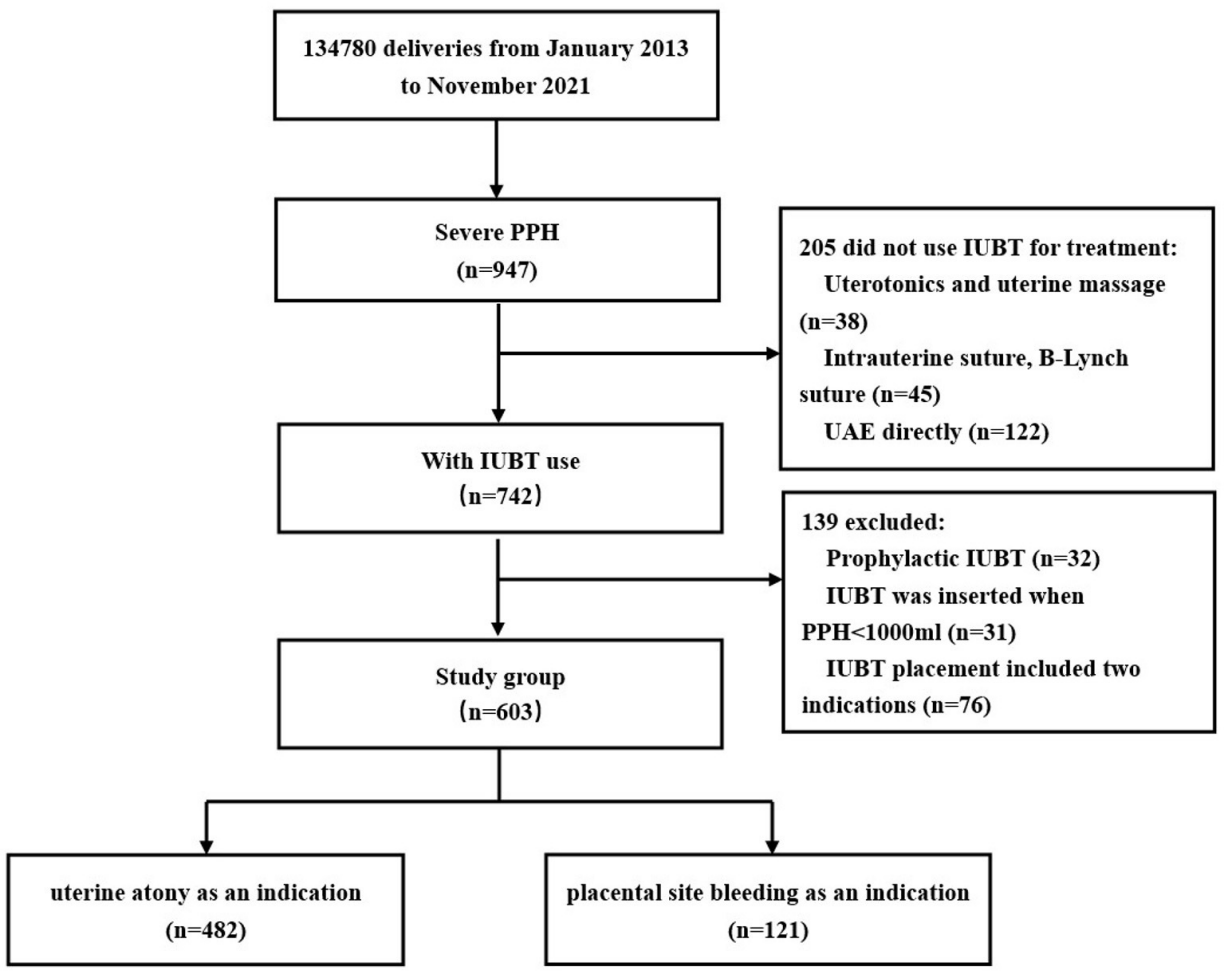
Flowchart. IUBT, intrauterine balloon tamponade; PPH, postpartum hemorrhage; UAE, uterine artery embolization.

During the study time frame (9 years), a total of 134,780 deliveries were performed at our institution, including 742 women with IUBT for PPH, of which 139 were excluded. Finally, 603 women were included in the study, with 482 (79.9%) having uterine atony as the main indication and 121 (20.1%) having placental site bleeding as the main indication (Figure 1). During the study period, there were 18 women with PPH ≥ 3,000 ml, 10 of whom had uterus hysterectomy, and there were no maternal deaths due to PPH or related complications.

Maternal demographics, obstetric baseline, and IUBT-related data are presented in Table 1. Compared with the group with uterine atony, the group with placental site bleeding had a greater proportion of parous women (57.9 vs. 42.9%, *p* = 0.004) and premature births (14.9 vs. 6.0%, *p* = 0.002), but the rate of induction of labor was relatively low (20.7 vs. 40.5%, *p* < 0.002). The proportion of two or more uterotonics applied was higher in the group with uterine atony compared with the group with placental site bleeding (91.5 vs. 82.6%, *p* = 0.009). No significant differences were seen in the maternal age, pre-pregnancy BMI, multiple gestation, history of PPH, antenatal hemoglobin, delivery mode, surgical hemostatic before IUBT, time from birth to IUBT, IUBT volume, duration of IUBT, or postpartum SI between the two groups (both *p* ≥ 0.05).

**Table 1:** Demographic, obstetric, and IUBT-related data were grouped according to IUBT indications.

	**Uterine atony**	**Placental site bleeding**	***P-*value**
	**(*n* = 482)**	**(*n* = 121)**	
Maternal age (years)	32.5 ± 4.5	33.4 ± 4.7	0.143
Pre-pregnancy BMI (kg/m^2^)	23.4 ± 4.1	23.7 ± 4.3	0.312
Nulliparous	275 (57.1)	51(42.1)	0.004
Multiple gestation	110 (22.8)	22 (18.2)	0.325
Premature birth	29 (6.0)	18 (14.9)	0.002
Induction of labor	195 (40.5)	25 (20.7)	<0.001
History of PPH	49 (10.2)	11(9.1)	0.741
Antenatal hemoglobin (mg/dl)	11.5 ± 1.4	11.6 ± 1.5	0.883
**Delivery mode**			
Cesarean	213 (44.2)	50 (42.1)	0.569
Vaginal	269 (55.8)	71 (57.9)	
**Surgical hemostatic before IUBT**			
None	330 (68.4)	79 (65.3)	0.154
Intrauterine suture	130 (27.0)	31 (25.6)	
B-Lynch suture	22 (4.6)	11 (9.1)	
**Uterotonics before UBT**			
0 or 1	43 (8.9)	21 (17.4)	0.009
≥2	439 (91.1)	100 (82.6)	
Time from birth to IUBT (min)	45 (15–80)	45 (15–75)	0.082
Duration of IUBT (h)	18.3 ± 5.6	18.7 ± 8.5	0.442
**IUBT volume (ml)**			
<250	24 (5.0)	8 (6.6)	0.371
250–350	432 (89.6)	103 (85.1)	
≥350	26 (5.4)	10 (8.3)	
Postpartum SI			
<1	30 (6.2)	13 (10.7)	0.145
1–1.4	340 (70.6)	86 (71.1)	
≥1.5	112 (23.2)	22 (18.2)	

*The values are given in the form of a number (percentage) or mean ± SD or median and interquartile range*.

*The Chi-square test were used for categorical variables. The independent sample t-tests or Mann-Whitney U-tests were used for continuous variables*.

*BMI, body mass index; IUBT, intrauterine balloon tamponade; PPH, postpartum hemorrhage*.

Univariate analysis was performed with IUBT indication as a covariate and PPH outcomes as a dependent variable. The incidence of transfusion ≥ 4 units of PRBCs was significantly higher in the group with placental site bleeding than in the group with uterine atony (34.7 vs. 16.8%, *p* < 0.001). Similar results were observed for EBL ≥ 300 ml after IUBT (43.8 vs. 20.0%, *p* < 0.001) and UAE or hysterectomy (8.3 vs. 3.5%, *p* = 0.029) (Table 2).

**Table 2:** Outcomes of women with PPH treated by IUBT due to uterine atony or placental site bleeding.

	**Uterine atony**	**Placental site bleeding**	***P-*value**
	**(*n* = 482)**	**(*n* = 121)**	
**Transfusion of** **≥4 units of PRBCs**			
Yes	81 (16.8)	42 (34.7)	<0.001
No	401 (83.2)	79 (65.3)	
**EBL** **≥300 ml after IUBT**			
Yes	99 (20.5)	53(43.8)	<0.001
No	383 (79.5)	68(56.2)	
**UAE or hysterectomy**			
Yes	17 (3.5)	10 (8.3)	0.029
No	465 (96.5)	111 (91.7)	

*The values are given in the form of a number (percentage)*.

*The Chi-square test were used for categorical variables*.

*CI, confidence interval; EBL, estimated blood loss; IUBT, intrauterine balloon tamponade; OR, odds ratio; PPH, postpartum hemorrhage; PRBCs, packed red blood cell; UAE, uterine artery embolization*.

Adjusting for confounding factors by multivariate logistic regression analysis, in general, PPH outcomes were worse in those who underwent IUBT for placental site bleeding. Placental site bleeding increased the risk of transfusion ≥ 4 units of PRBCs (aOR 2.47, 95% CI 1.32–3.98), EBL ≥ 300 ml after IUBT (aOR 3.78, 95% CI 2.22–5.33), and UAE or hysterectomy (aOR 2.52, 95% CI 1.20–6.01) when compared to uterine atony. The higher antenatal hemoglobin values were negatively correlated with the incidence of transfusion ≥ 4 units of PRBCs (aOR 0.60, 95% CI 0.41–0.83) and UAE or hysterectomy (aOR 0.71, 95% CI 0.32–0.90). Different cut-off values were used to stratify the IUBT volume. We observed that the risk of transfusion ≥ 4 units of PRBCs (aOR 3.43, 95%CI 1.76–6.52) and EBL ≥ 300 ml (aOR 2.32, 95%CI 1.27–3.98) was significantly increased when IUBT volume ≥ 350 ml. Interestingly, as the prolonged duration of IUBT increased, the risk of EBL ≥ 300 ml was significantly increased (aOR 1.27, 95%CI 1.04–1.62). As in clinical practice, an SI value ≥ 1.5 was significantly associated with an increased risk of transfusion ≥ 4 units of PRBCs (aOR 3.43, 95%CI 1.76–6.52) and UAE or hysterectomy (aOR 2.29, 95%CI 1.06–4.14) (Tables 3–5).

**Table 3:** Risk factors for the prevalence of transfusion ≥ 4 units of PRBCs.

	**Univariate**		**Multivariate**	
	**OR (95% CI)**	***P-*value**	**aOR (95% CI)**	***P-*value**
**IUBT indications**				
Uterine atony	Reference	–	Reference	–
Placental site bleeding	2.63 (1.69–4.10)	<0.001	2.47 (1.32–3.98)	<0.001
Maternal age	1.03 (0.94–1.05)	0.791	–	–
Multiple gestation	1.56 (0.82–2.89)	0.411	–	–
Antenatal hemoglobin(mg/dl)	0.62 (0.41–0.87)	0.023	0.60 (0.41-0.83)	0.019
**Delivery mode**				
Vaginal	Reference	–	–	–
Cesarean	1.03 (0.74–1.44)	0.792	–	–
Time from birth to IUBT (min)	1.02 (0.91–1.03)	0.834	–	–
Duration of IUBT (h)	1.13 (0.66-1.87)	0.079	–	–
**IUBT volume (ml)**				
250–350	Reference	–	Reference	–
<250	1.28 (0.81–1.88)	0.523	–	–
≥350	3.58 (1.88–6.79)	<0.001	3.43 (1.76–6.52)	<0.001
**Postpartum SI**				
<1	Reference	–	Reference	–
1–1.4	1.69 (1.10–2.32)	0.011	1.72 (1.28–2.57)	0.009
≥1.5	3.27 (1.82–4.55)	<0.001	3.43 (2.22–4.89)	<0.001

*CI, confidence interval; EBL, estimated blood loss; OR, odds ratio; PRBCs, packed red blood cell; IUBT, intrauterine balloon tamponade; PPH, postpartum hemorrhage; UAE, uterine artery embolization; SI, shock index*.

**Table 4:** Risk factors for the prevalence of EBL ≥ 300 ml after IUBT.

	**Univariate**		**Multivariate**	
	**OR (95% CI)**	***P-*value**	**aOR (95% CI)**	***P-*value**
**IUBT indications**				
Uterine atony	Reference	–	Reference	–
Placental site bleeding	3.02 (1.98–4.60)	<0.001	3.78 (2.22–5.33)	<0.001
Maternal age	0.97 (0.72–1.18)	0.329	–	–
Multiple gestation	1.67 (0.83–2.76)	0.222	–	–
Antenatal hemoglobin(mg/dl)	0.89 (0.66–1.09)	0.143	–	–
**Delivery mode**				
Vaginal	Reference	–	–	–
Cesarean	0.66 (0.37–1.27)	0.087	–	–
Time from birth to IUBT (min)	0.93 (0.67–1.12)	0.286	–	–
Duration of IUBT (h)	1.24 (1.03–1.78)	0.033	1.27 (1.04-1.62)	0.028
**IUBT volume (ml)**
250–350	Reference	–	Reference	–
<250	1.28 (0.81–1.93)	0.412	–	–
≥350	2.26 (1.21–3.79)	0.019	2.32 (1.27–3.98)	0.013
**Postpartum SI**				
<1	Reference	–	–	–
1–1.4	1.24 (0.59–2.07)	0.482	–	–
≥1.5	1.58 (0.83–2.79)	0.369	–	–

*CI, confidence interval; EBL, estimated blood loss; OR, odds ratio; PRBCs, packed red blood cell; IUBT, intrauterine balloon tamponade; PPH, postpartum hemorrhage; UAE, uterine artery embolization; SI, shock index*.

**Table 5:** Risk factors for the prevalence of UAE or hysterectomy.

	**Univariate**		**Multivariate**	
	**OR (95% CI)**	***P-*value**	**aOR (95% CI)**	***P-*value**
**IUBT indications**				
Uterine atony	Reference	–	Reference	–
Placental site bleeding	2.46 (1.10–5.53)	0.029	2.52 (1.20–6.01)	0.023
Maternal age	1.14 (0.72–1.52)	0.721	–	–
Multiple gestation	1.22 (0.78–1.62)	0.182	–	–
Antenatal hemoglobin(mg/dl)	0.73 (0.34-0.92)	0.033	0.71 (0.32-0.90)	0.029
**Delivery mode**				
Vaginal	Reference	–	–	–
Cesarean	1.29 (0.92–2.04)	0.132	–	–
Time from birth to IUBT (min)	1.01 (0.65–1.99)	0.826	–	–
Duration of IUBT (h)	0.92 (0.31-1.40)	0.423	–	–
**IUBT volume (ml)**				
250–350	Reference	–	Reference	–
<250	0.96 (0.49–1.34)	0.780	–	–
≥350	1.11 (1.03–1.68)	0.038	1.09 (1.01–1.47)	0.043
**Postpartum SI**				
<1	Reference	–	Reference	–
1–1.4	1.43 (0.82–3.01)	0.108	–	–
≥1.5	2.33 (1.07–4.66)	0.032	2.29 (1.06–4.14)	0.040

*CI, confidence interval; EBL, estimated blood loss; OR, odds ratio; PRBCs, packed red blood cell; IUBT, intrauterine balloon tamponade; PPH, postpartum hemorrhage; UAE, uterine artery embolization; SI, shock index*.

## Discussion

### Main Findings

This retrospective cohort study demonstrated that the therapeutic effect of IUBT for severe PPH was not the same for different indications. We also found a significant association between antenatal hemoglobin, IUBT volume, duration of IUBT, postpartum SI, and PPH outcomes.

### Interpretation

We found that in PPH patients, IUBT performed in the placental site bleeding group was more likely to have adverse PPH outcomes compared to the uterine atony group. Although IUBT was originally proposed by Bakri to be used in PPH patients with placenta previa or low-lying placenta (8), the sample size was only 5, and the study efficacy was limited. Previous studies have shown that placenta accreta spectrum (PAS) was negatively associated with the success of IUBT (12, 13). According to Liu et al. (14), IUBT was only 54.8% successful in cases with previa abnormalities. Our study indicates that perhaps IUBT for PPH due to placental abnormalities is not the best management measure given the high incidence of adverse outcomes after placement. In cases with placental abnormalities, the placenta was often unable to detach from the uterine wall, and manual detachment may result in active bleeding due to damage to the myometrium. Whether the compression force of the balloon alone to stop bleeding can achieve the ultimate therapeutic effect remains to be further investigated. However, it is worth mentioning that IUBT is a simple and rapid way to stop bleeding. IUBT can be used as a temporary hemostatic measure until other treatment measures are prepared, even if the final treatment is ineffective.

The present study found a correlation between higher prenatal hemoglobin levels and good outcomes in PPH patients treated with IUBT, which was consistent with previous research (15). To reduce the morbidity associated with PPH, the Royal College of Obstetricians and Gynecologists published guidelines in 2016 recommending that antenatal anemia be investigated and treated appropriately (2). A higher prenatal hemoglobin value means that the mother has a greater reserve capacity for blood loss, which is an important guideline for managing PPH.

SI aids in predicting the severity of early hypovolemic states and determining the need for transfusion or surgical treatment in cases of massive blood loss (16). SI has always been equally important in determining the need for intervention in PPH, and it plays a more important role than HR and BP indicators (17). According to the findings of this study, elevated SI values are an important predictor of postpartum transfusion and the need for additional invasive procedures.

Ji et al. suggested in a retrospective cohort study of 289 individuals (15) that IUBT volume > 350 ml was associated with adverse PPH outcomes. Similar results were obtained in our study. A reasonable explanation is that a larger IUBT volume may suggest a softer uterus or require more pressure to compress the uterine cavity to stop the bleeding. The conclusion that some studies (18) have reported greater amounts of fluid required to fill the balloon and a higher rate of IUBT failure in the case of multiple and twin births validates the findings mentioned above to some extent. However, it is worth mentioning that the direct cause of the adverse PPH outcomes was the patient's underlying condition rather than the more fluid. IUBT volume can be used as a proxy when it is not possible to quantify the degree of uterine softness or the required pressure to stop bleeding in patients with PPH.

To our knowledge, no previous studies have demonstrated the impact of IUBT duration time on PPH outcomes. Although the instructions recommend placement for no longer than 24 h, various guidelines do not suggest how long IUBT placement should be retained. The present study showed a positive correlation between prolonged IUBT duration time and EBL ≥ 300 ml. Alouini (19) suggests that the additional IUBT placement time may be due to persistent uterine bleeding without other techniques to control the bleeding. We believe well-designed prospective studies are required if precise conclusions are desired.

### Strengths and Limitations

First, thanks to the high volume of deliveries at our institution, this study included 603 participants, which is a larger sample size than any previous one. Second, we refined the PPH outcomes after IUBT into EBL > 300 ml, transfusion > 4 units PRBCs, and UAE or hysterectomy, which allowed a more adequate assessment of maternal changes after IUBT. The main limitation of this study is that it is a retrospective study because PPH treatment is often performed in emergency situations and it is difficult to perform controlled trials. Bias in data quality and sample selection is difficult to avoid. Also, the etiology of PPH in patients sometimes overlaps, e.g., uterine atony may be secondary to placental abnormalities, the main cause of which depends mainly on the clinician's determination.

## Conclusion

In this study, our data suggest that IUBT is a relatively reliable treatment for PPH, but its therapeutic efficacy varies depending on the indication for placement. Patients with PPH due to placental site bleeding should receive more attention in clinical practice. Prenatal hemoglobin and SI are good predictors of PPH outcome, and clinicians should actively correct anemia and closely monitor SI values in patients with PPH. The causal relationship between the IUBT volume, the IUBT duration time, and the PPH outcomes still needs to be corroborated by a large number of controlled trials.

## Data Availability Statement

The raw data supporting the conclusions of this article will be made available by the authors, without undue reservation.

## Ethics Statement

The studies involving human participants were reviewed and approved by GKLW 2021–55. The Ethics Committee waived the requirement of written informed consent for participation.

## Author Contributions

This study was designed by CX, XChe, and LW, who also analyzed and interpreted the data. CX and YC gathered information and wrote the manuscript. All authors discussed the findings, contributed to the article, and signed off on the final draft.

## Funding

This study was supported by Chinese Academy of Medical Sciences Research Unit (No. 2019RU056), Shanghai Jiao Tong University, CAMS Innovation Fund for Medical Sciences (CIFMS) (No. 2019-I2M-5-064), and Shanghai Municipal Key Clinical Specialty, Shanghai, China (No. shslczdzk01802).

## Conflict of Interest

The authors declare that the research was conducted in the absence of any commercial or financial relationships that could be construed as a potential conflict of interest.

## Publisher's Note

All claims expressed in this article are solely those of the authors and do not necessarily represent those of their affiliated organizations, or those of the publisher, the editors and the reviewers. Any product that may be evaluated in this article, or claim that may be made by its manufacturer, is not guaranteed or endorsed by the publisher.
